# Distinctive Correspondence Between Separable Visual Attention Functions and Intrinsic Brain Networks

**DOI:** 10.3389/fnhum.2018.00089

**Published:** 2018-03-12

**Authors:** Adriana L. Ruiz-Rizzo, Julia Neitzel, Hermann J. Müller, Christian Sorg, Kathrin Finke

**Affiliations:** ^1^Department of General and Experimental Psychology, Ludwig-Maximilians-Universität München, Munich, Germany; ^2^Graduate School of Systemic Neurosciences, Ludwig-Maximilians-Universität München, Munich, Germany; ^3^Department of Neuroradiology, Klinikum Rechts der Isar, Technische Universität München, Munich, Germany; ^4^School of Psychological Science, Birkbeck College, University of London, London, United Kingdom; ^5^Hans-Berger Department of Neurology, Friedrich Schiller University Jena, Jena, Germany

**Keywords:** functional connectivity, intrinsic brain networks, resting-state fMRI, top-down control, visual attention, visual processing speed

## Abstract

Separable visual attention functions are assumed to rely on distinct but interacting neural mechanisms. Bundesen's “theory of visual attention” (TVA) allows the mathematical estimation of independent parameters that characterize individuals' visual attentional capacity (i.e., visual processing speed and visual short-term memory storage capacity) and selectivity functions (i.e., top-down control and spatial laterality). However, it is unclear whether these parameters distinctively map onto different brain networks obtained from intrinsic functional connectivity, which organizes slowly fluctuating ongoing brain activity. In our study, 31 demographically homogeneous healthy young participants performed whole- and partial-report tasks and underwent resting-state functional magnetic resonance imaging (rs-fMRI). Report accuracy was modeled using TVA to estimate, individually, the four TVA parameters. Networks encompassing cortical areas relevant for visual attention were derived from independent component analysis of rs-fMRI data: visual, executive control, right and left frontoparietal, and ventral and dorsal attention networks. Two TVA parameters were mapped on particular functional networks. First, participants with higher (vs. lower) visual processing speed showed lower functional connectivity within the ventral attention network. Second, participants with more (vs. less) efficient top-down control showed higher functional connectivity within the dorsal attention network and lower functional connectivity within the visual network. Additionally, higher performance was associated with higher functional connectivity between networks: specifically, between the ventral attention and right frontoparietal networks for visual processing speed, and between the visual and executive control networks for top-down control. The higher inter-network functional connectivity was related to lower intra-network connectivity. These results demonstrate that separable visual attention parameters that are assumed to constitute relatively stable traits correspond distinctly to the functional connectivity both within and between particular functional networks. This implies that individual differences in basic attention functions are represented by differences in the coherence of slowly fluctuating brain activity.

## Introduction

Separable visual attention functions are assumed to rely on distinct but interacting neural mechanisms (Posner and Petersen, 1990; Desimone and Duncan, 1995; Bundesen et al., 2005). The computational “theory of visual attention” (TVA, Bundesen, 1990) permits a set of independent parameters to be estimated that reflect individuals' attentional capacity (i.e., visual processing speed and short-term memory storage capacity) and selectivity (i.e., top-down control and spatial laterality). These TVA parameters have been suggested to constitute traits that characterize individuals' speed and efficiency of attentional selection processes (Finke et al., 2005). The relationship between these parameters and the basic organization of the brain has mainly been examined in local lesion studies. Thus, for example, reduced visual processing speed has been associated with temporoparietal junction (Peers et al., 2005) and lateral thalamic non-traumatic lesions (Kraft et al., 2015), as well as with a parietal white-matter reduction in posterior cortical atrophy (Neitzel et al., 2016). A lateral spatial bias has been documented following medial thalamic lesions (Kraft et al., 2015) as well as asymmetric parietal hypometabolism induced by early Alzheimer's disease (Sorg et al., 2012). Studies on the neural organization of these parameters in the healthy brain are comparatively rare. A structural connectivity analysis revealed visual short-term memory (VSTM) capacity to be associated with the organization of the superior longitudinal and inferior fronto-occipital fasciculi (Chechlacz et al., 2015). Top-down control was found to be associated with task-related functional connectivity among parietal areas (Vossel et al., 2016). Taken together, these findings imply that TVA parameters closely reflect the integrity of attention-relevant brain areas and their connections, including their functional interactions. It remains, however, unknown whether and how these parameters map onto functional networks overlapping those attention-relevant areas.

Functional networks that include regions relevant for visual attention have been identified based on their intrinsic functional connectivity (FC) (Fox et al., 2006; Smith et al., 2009; Allen et al., 2011; Yeo et al., 2011; Raichle, 2015). Intrinsic FC represents the correlation, among different brain regions, of infra-slowly (i.e., 0.01–0.1 Hz) ongoing blood oxygenation level dependent (BOLD) signal intensity fluctuations obtained from resting-state functional magnetic resonance imaging (fMRI) (Fox and Raichle, 2007; Raichle, 2011). Such fluctuations reflect the dynamics of slowly propagating activity including cortical neuronal excitability (Wu et al., 2008; Matsui et al., 2016), linked with faster oscillatory activity by cross-frequency phase-amplitude coupling (Mantini et al., 2007; He et al., 2010; Brookes et al., 2011; Hipp et al., 2012). Intrinsic FC provides relevant information on both brain-evoked activity (Mennes et al., 2010) and behavior (Markett et al., 2014; Rosenberg et al., 2016, 2017). Crucially, the brain networks identified through intrinsic FC are stable both within (Zuo et al., 2010) and across subjects (Damoiseaux et al., 2006; De Luca et al., 2006), and largely correspond to structural connectivity (Damoiseaux and Greicius, 2009; Honey et al., 2009). These characteristics collectively suggest the possibility of a distinctive correspondence between specific, separable visual attention functions and particular intrinsic brain networks.

Here we examined whether and how independent visual attention parameters obtained from modeling using TVA are mapped onto distinct functional networks derived from intrinsic FC. Crucially, to avoid potential confounding by structural integrity or visual attention changes inherent in patient or developing populations, we examined an age-homogeneous group of healthy participants. Moreover, following the neural interpretation of TVA (Bundesen et al., 2005), we focused on networks that comprise brain regions relevant for visual attention (for a review, see Parks and Madden, 2013). White matter pathways might anatomically constrain functional network connectivity (Parks and Madden, 2013), albeit not in a one-to-one fashion (Damoiseaux and Greicius, 2009). Accordingly, based on the results of previous TVA-based studies relating individual variability in attention functions to variability in structural connectivity (e.g., Chechlacz et al., 2015), we expected to find a positive association between TVA parameter estimates and intrinsic FC.

## Materials and methods

### Participants

Thirty-two healthy young subjects (25–27 years old) participated in this study. The “Klinikum rechts der Isar's” Ethics Committee approved the study, which was conducted in agreement with the Declaration of Helsinki, and all participants gave written informed consent and were paid for their participation. All participants underwent BOLD-fMRI during rest and TVA-based assessment in separate sessions conducted on the same day (though one participant did not perform the TVA partial-report task and thus had to be excluded from the analyses). All had a normal or corrected-to-normal visual acuity and normal color vision. Before visual attention and MRI examination, participants were assessed for global cognitive functioning by trained psychologists using a short version of the German Wechsler Adult Intelligence scale-III (WAIS-III) (Von Aster et al., 2006), permitting computation of Full-Scale IQ. Demographic information is listed in Table 1. Males and females did not differ in any of the demographic variables.

**Table 1:** Demographic variables.

**Demographic variable**	**Entire sample (*n* = 31)**	**Females (*n* = 14)**	**Males (*n* = 17)**	***p*-value**
Age [years]	26.56 ± 0.55	26.61 ± 0.55	26.52 ± 0.56	0.680
Education [years]	11.55 ± 1.59	11.50 ± 1.56	11.59 ± 1.66	0.881
Intelligence [IQ]	99.94 ± 11.64	100.57 ± 8.55	99.41 ± 13.93	0.788

*Mean ± standard deviations are shown*.

### Parametric assessment and estimation of visual attention functions

#### General procedure

**Figure 1:**
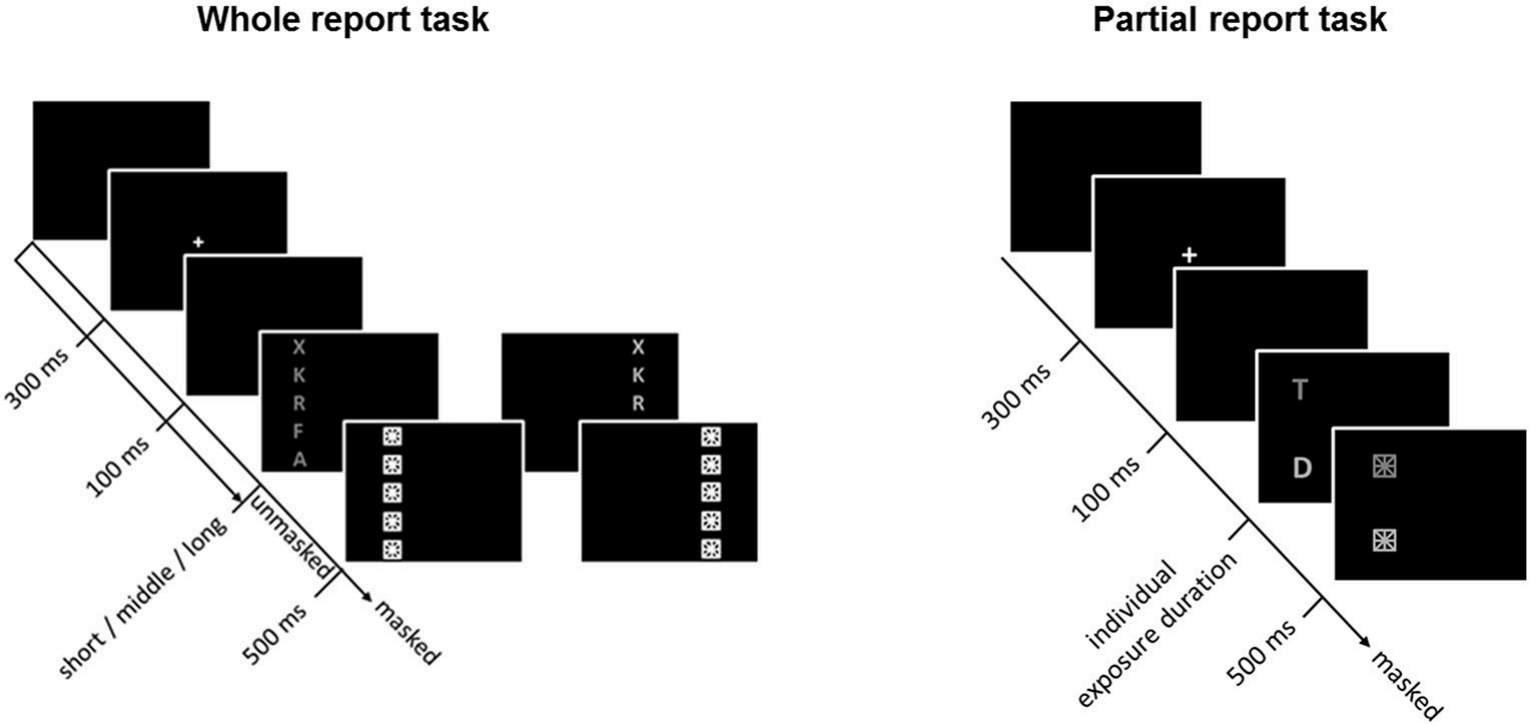
Whole- (**left**) and partial-report (**right**) tasks used to assess and estimate visual attention functions. In the partial-report task, targets (T) are presented in red, and distracters (D) in green.

The general TVA-based procedure for assessing visual attention functioning has been described in detail elsewhere (e.g., Finke et al., 2015). Briefly, to assess visual attention functions, participants performed, in a balanced order, whole- and partial-report tasks that lasted ~0.5 h each. Within a trial, a central white cross (0.3° visual angle) appeared for 300 ms, followed by a 100-ms gap after which the task-relevant stimuli were presented (Figure 1). Stimuli comprised of red or green letters (0.5° high × 0.4° wide) randomly chosen from a pre-specified set (“ABEFHJKLMNPRSTWXYZ”). Letters were generally terminated by masks (each composed of a square with a star inside), effectively overwriting iconic memory traces of the stimulus array (see below). Note though that trials without post-display masks were introduced in the whole-report task in order to increase the variability of “effective” exposure times (by allowing for an additional component of iconic memory buffering; Sperling, 1960) and thus ensure reliable and valid TVA parameter estimation. Stimuli were presented on a 17-inch monitor (1,024 by 1,280 pixel screen resolution, 60-Hz refresh rate), in a dimly lit room.

#### Visual attention capacity parameters

Capacity parameters were derived from report accuracy in the whole-report task (Figure 1, left), in which participants were instructed to report all letters they were fairly sure they had seen. First, in a pretest (24 trials), one individualized exposure duration was determined as the presentation time required to report one letter on average over several trials correctly. Shorter and longer exposure durations were then determined based on that value. Next, the three durations were used to present stimuli either unmasked or immediately followed by masking stimuli, thus resulting in six effective exposure durations (for more details, see Finke et al., 2015). The average short, intermediate, and long exposure durations were, respectively, 45.17 (*SD* = 7.0), 82.23 (*SD* = 17.26), and 164.90 (*SD* = 33.40) ms. The task consisted of 192 trials presented in 4 blocks of 48 trials each. Within each block, trials were randomized and presented equally often under 12 conditions (2 masking conditions [no masks, post-display masks] × 3 exposure durations × 2 hemifields). Performance accuracy (i.e., the number of letters reported correctly) was measured as a function of the effective exposure duration. Based on TVA, an exponential growth function was used to model the probability of selecting an object (Bundesen, 1990; Kyllingsbaek, 2006). The slope of the exponential curve at the minimum effective exposure duration *t0* (for masked displays) reflects the processing rate *C*—or number of elements processed per second—and the asymptote indicates the VSTM storage capacity—or the maximum number of items that can be simultaneously represented in VSTM. The effective additional exposure duration in unmasked displays (or parameter *m*μ) attributable to iconic memory buffering was also determined to validly estimate parameters *C* and *K*. Even though *m*μ was of no further interest in our study, it was necessary to estimate because, in unmasked displays, retention of visual traces in iconic memory allows for prolonged information processing (Finke et al., 2015).

#### Visual attention weighting parameters

Attentional weighting parameters were derived from report accuracy in the partial-report task (Figure 1, right), in which participants had to report targets (red letters) and ignore distractors (green letters). On each trial, (a) a single target, (b) a target and a distractor, or (c) two targets were presented horizontally or vertically at the corners of an imaginary square (for more details, see Finke et al., 2015). As in the whole-report task, the individual exposure duration was determined in a pretest (32 trials) as the duration at which the participant reported single targets with 80% accuracy. The average exposure duration was 91.50 ms (*SD* = 23.42). The task consisted of 6 blocks of 48 trials each (i.e., 288 trials in total). In contrast to the whole-report task, the stimuli were always followed by a mask under 16 conditions (4 single target conditions, 8 target plus distractor conditions, and 4 dual target conditions). From the probabilities of target report, attentional weights were separately derived for targets and distractors, and for each visual hemifield, based on TVA. The selectivity of attentional weighting, or top-down control α, was estimated as the ratio of the attentional weights allocated to targets to the weights assigned to distractors. Lower α values would then indicate high selectivity or preference for targets (i.e., more efficient top-down control), whereas higher values would indicate less selective processing. In turn, the spatial distribution of attention across visual hemifields, or spatial laterality *w*_*lat*_, was defined as *w*_*left*_/ (*w*_*left*_ + *w*_*right*_), where *w*_*left*_ indicates the attentional weight allocated to the left visual hemifield and *w*_*right*_ the attentional weight allocated to the right visual hemifield. A value of 0.5 indicates balanced weighting, whereas values above or below 0.5 would be indicative of, respectively, left- or right-ward spatial laterality (Finke et al., 2005).

### Resting-state fMRI

#### Imaging data acquisition

Imaging data were acquired on a 3T MR scanner (Achieva TX, Philips, Netherlands) with an 8-channel phase-array head coil. Participants lay comfortably with their heads surrounded by soft foams to reduce head motion. Before starting the functional data acquisition, participants were instructed to close their eyes but avoid falling asleep (i.e., resting state), and we checked with them at the end of the sequence that they had actually stayed awake. Functional data were collected across 10 min 52 s during resting state, and comprised 250 T2^*^-weighted volumes using a gradient-echo echo-planar sequence: *TR* = 2,608 ms; *TE* = 35 ms; flip angle = 90°; FOV = 230 mm^2^; matrix size = 64 × 63, 41 slices with 3.58 mm thickness and no interslice gap; reconstructed voxel size = 3.59 mm isotropic. Structural data were obtained from a T1-weighted magnetization-prepared rapid-acquisition gradient echo (MPRAGE) sequence: *TR* = 7.71 ms; *TE* = 3.93 ms; flip angle = 15°; field of view (FOV) = 256 mm^2^; matrix = 256 × 256, 180 slices; voxel size = 1 mm^3^.

#### Imaging data preprocessing

The imaging data were preprocessed using the Data Processing Assistant for Resting-State fMRI (DPARSF; Chao-Gan and Yu-Feng, 2010), a toolbox in MATLAB (R2013a, version 8.1.0.604; The Mathworks Inc.; Natick, MA, USA). Briefly, the preprocessing included realignment, reorientation to the AC-PC axis of functional and structural images; segmentation of the structural T1-weighted image and co-registration of the segmented T1-weighted and the T2*-weighted functional images. No participant had to be excluded based on excessive head motion, which was defined as cumulative translation or rotation larger than 3 mm or 3°, or mean point-to-point translation or rotation greater than 0.15 mm or 0.1°. Six head motion parameters, as well as white matter, CSF, and global signals were entered as nuisance covariates and regressed out from the functional data. Next, functional images were normalized into the Montreal Neurological Institute (MNI) space using unified segmentation of T1 image (Ashburner and Friston, 2005), and resampled to 2-mm isotropic voxel size to keep the highest resolution possible. The normalized images were then smoothed using a 4-mm full-width-at-half-maximum (FWHM) Gaussian kernel.

#### Independent component and dual regression analyses

Preprocessed data were temporally concatenated and analyzed by probabilistic independent component analysis (ICA) as implemented in FSL (version 5.0.7) using MELODIC version 3.14 (Beckmann and Smith, 2004; Smith et al., 2004). A low dimensionality (i.e., 20 independent components) was chosen to decompose the data into more spatially extended components reflecting intrinsic brain networks (Smith et al., 2009).

To obtain estimates of independent components for each participant, we performed a dual regression analysis (Beckmann et al., 2009; Filippini et al., 2009) using the group-independent components generated in the group ICA as input. Dual-regression analysis permits quantifying, for each subject, the FC of each voxel with each group-independent component while controlling for all other components (some of which represent artifacts) (Smith et al., 2014). Crucially for our study, dual-regression analysis is superior in detecting individual variability in FC compared to traditional approaches, such as seed-based analysis (Smith et al., 2014). Finally, FSL's *randomize* permutation-testing tool, based on 500 permutations and a *p*-value of 0.05, was used to obtain group spatial maps.

The individual networks for each participant included voxel-wise Z-scores or standardized parameter estimates (by the residual within-subject noise) obtained from the second stage of the dual regression (for details, e.g., Smith et al., 2014). In other words, each map contained voxel-wise information on the particular contribution to an independent component while controlling for the influence of its contribution to all the other components (Filippini et al., 2009; Smith et al., 2014). Thus, for each participant, we obtained 20 individual maps (one for each component), with the Z-score of every voxel, within each map, indicating how closely that voxel's time course resembled that of the respective group component. These individual voxel-wise Z-maps were further used for group statistics, in which group differences could manifest in any brain region belonging to the independent component, irrespective of whether or not that region is *typically* included in the brain network that the independent component represents (Smith et al., 2014).

#### Selection of intrinsic brain networks for further statistical analysis

The particular choice of networks on which we focused our analyses was based on both the neural interpretation of TVA (Bundesen et al., 2005) and the standard templates for intrinsic brain networks reported in the resting-state fMRI literature (e.g., Allen et al., 2011; Yeo et al., 2011). However, to establish a correspondence between distinct visual attention parameters and distinct intrinsic brain networks, we first needed to ensure that the relative independence among the networks was comparable to that amongst the different TVA parameters. For this reason, we chose ICA over, for instance, a seed-based approach: as a multivariate approach, ICA yields a set of statistically independent sources or components (Beckmann and Smith, 2004); and as a data-driven approach, it can remove the noise (e.g., both physiological and scanner-related) from the data (Zuo et al., 2010).

To select independent components representing intrinsic brain networks assumed to play a role in visual attention, we first identified relevant intrinsic networks by referring to typical networks described previously. In detail, to automatically select independent components reflecting intrinsic networks, we conducted multiple spatial cross-correlations with templates derived from FC based on resting-state fMRI of 1,000 healthy subjects (Yeo et al., 2011), in which a 7-network parcellation of the cortex was found robust, including visual, dorsal and ventral attention, and frontoparietal networks. It should be noted that the labeling of these networks—though fitting in the context of attention research—is somewhat arbitrary, as these networks are also involved in other cognitive functions (Smith et al., 2009), that is, there is no one-to-one mapping between intrinsic networks and function. After that, we chose the networks that best covered regions proposed by neural TVA to contribute to visual attention functions (i.e., frontal, parietal, limbic, and occipital; Bundesen et al., 2005), in particular: the visual, executive control, lateralized frontoparietal, and ventral and dorsal attention networks. To be independent of the special parcellation approach used by Yeo and colleagues for intrinsic networks (i.e., clustering), we considered it reasonable to compare our spatial maps with network templates obtained using ICA. Thus, we conducted further spatial cross-correlations but with intrinsic brain network templates derived from an ICA approach based on the resting-state fMRI data of 603 healthy subjects (Allen et al., 2011). We found the chosen networks to exhibit the greatest overlap with frontoparietal and occipital-visual networks (i.e., IC60, IC72, IC55, IC34, IC64, and IC27 of Allen et al., 2011) that have been related to attention functions previously (e.g., Corbetta and Shulman, 2002; Fox et al., 2006; Dosenbach et al., 2007, 2008; Vincent et al., 2008; Smith et al., 2009; Finke et al., 2015), thus confirming our selection of attention-relevant brain networks. Note that ICA-derived spatial maps can have a larger extension and include more regions than those classically associated with a specific network (e.g., Smith et al., 2014), without compromising the reliability of the method (e.g., Zuo et al., 2010).

### Statistical analysis

#### Intra-network differences in functional connectivity between performance groups

To examine for intra-network differences in functional connectivity, we took the individual versions of the previously selected networks from the results of the second stage of the dual-regression analysis (i.e., temporal regression; for details, e.g., Smith et al., 2014). First, based on the individual TVA parameter estimates, the group median was calculated separately for each of the parameters and used to split the sample into “high” and “low” performers (for parameters visual processing speed *C*, visual short-term memory capacity *K*, and top-down control α) and left- and right-preference (for parameter spatial laterality *w*_*lat*_). Next, we tested for differences in intrinsic FC in visual attention-relevant brain networks between the groups based on the median splits using Statistical Parametric Mapping, SPM8 (www.fil.ion.ucl.ac.uk/spm/software/spm8/). Specifically, using a second-level (i.e., group) general linear model, we predicted each voxel's intra-network FC (within each visual attention-relevant network) from TVA performance (i.e., performance-based subgroups of the variable of interest), controlling for the remaining three TVA parameters and for education and gender (i.e., variables of no interest).

Because our goal was to systematically examine whether and how distinct TVA parameters are independently mapped onto distinct functional networks of the healthy brain, we performed six (i.e., one for each brain network) two-sample *t*-tests for each TVA parameter of interest. In the general linear model, intra-network FC was predicted from 7 parameters (i.e., 24 degrees of freedom). We contrasted the first two parameters, which correspond to the two levels of the main variable “performance-based subgroup” (i.e., “high” and “low” performers). Within each group, the images included the individual network-specific Z-maps derived from dual regression; each voxel within each of these maps took on a value indicating how close its time course reflected the group component's time course. Results were corrected for multiple comparisons (*p* < 0.05 FWE-corrected at the cluster level, voxel-wise height threshold *p* < 0.001) and only results surviving the additional Bonferroni corrections at the network level (i.e., *p*_corr_ 0.05/6 networks = 0.0083) were considered further.

We chose a median split over a linear regression approach, for the following reasons. First, given the strong homogeneity of our sample regarding demographics, brain integrity, and behavior, we had no reasons to expect robust linear relationships between the voxel-wise intrinsic FC and the TVA parameters. Second, as previous TVA-based studies on small healthy samples had revealed significant differences between high and low performers in experimental manipulations (e.g., Finke et al., 2010) or brain measures (e.g., Wiegand et al., 2014), we wanted to keep our analyses and results comparable to these studies. Third, TVA parameters have been proposed to reflect relatively stable characteristics of a given individual (e.g., Finke et al., 2005; Habekost, 2015). Given this, we can assume that our median split-defined groups are random samples of “high” and “low” performers from the population. Finally, the independence of TVA parameters is given mathematically (Bundesen, 1990) and empirically (Habekost et al., 2014), which reduces the probability (Iacobucci et al., 2015) of Type I errors (Maxwell and Delaney, 1993). Note that, in our sample too, we did not find significant relationships between the different parameters: *p* > 0.072.

#### Inter-network differences in functional connectivity between performance groups

To examine for inter-network differences in functional connectivity, we took the results of the first stage of the dual regression (i.e., spatial regression; for details, e.g., Smith et al., 2014) and analyzed them using custom code written in MATLAB. For each participant, we correlated the time courses of the six independent components of interest and performed Fisher r-to-z transformation. Next, we tested whether the inter-network FC was significantly higher for “high” than for “low” performers. Finally, we examined whether intra-network FC correlated with inter-network FC.

## Results

### Visual attention parameters

Mean TVA parameter estimates for the entire sample, as well as separately for each performance and spatial laterality preference group are listed in Table 2. Note that for the spatial laterality parameter *w*_*lat*_, the group mean did not differ from the value of 0.5, which indicates optimally balanced attention [*t*_(30)_ = −0.569, *p* = 0.573]. Males and females did not differ significantly in any of the TVA parameter estimates (data not shown; all *p* > 0.179). The TVA parameters did not significantly correlate with each other (all *p* > 0.072; see Table 3 for pairwise correlations). Furthermore, except for a significant correlation between processing speed *C* and IQ (*r* = 0.37, *p* = 0.039), they also did not significantly correlate with any of the demographic variables in the entire sample (all other *p* > 0.135). The group medians for the four TVA parameters used to split the sample are listed in Table 2. Importantly, the resulting groups differed exclusively in the TVA parameter of interest and not in any of the other TVA parameters, education, age, IQ, or gender [*C*: *t*_(18.8)_ = 5.382, *p* < 0.0001, all other covariates: *p* > 0.150; *K*: *t*_(17)_ = 6.634, *p* < 0.00001, all other covariates: *p* > 0.108; α: *t*_(29)_ = −9.308, *p* < 0.00001, all other covariates: *p* > 0.184; *w*_*lat*_: *t*_(29)_ = −6.764, *p* < 0.00001, all other covariates: *p* > 0.191]. It is worth noting that only six participants (five males and one female) were always classified as “high” (three) or “low” (three) performers for *C, K*, and α. These participants did not differ in any demographic or TVA variable from the rest of the sample (*p* > 0.506). Thus, our participants have a distinct profile in terms of the different parameters, instead of exhibiting a more general, either “good” or “poor,” visual attention performance. Importantly, this corroborates the independence assumption maintained for the TVA parameters (e.g., Habekost et al., 2014) and indicates that the median split approach can be validly applied here.

**Table 2:** TVA parameter estimates.

**TVA parameter**	**Entire sample (*n* = 31)**	**High performance (*n* = 16)**	**Low performance (*n* = 15)**
Processing speed *C* (*Md* = 24.30)	25.89 ± 7.34	30.76 ± 7.05	20.70 ± 2.45
VSTM capacity *K* (*Md* = 2.83)	3.03 ± 0.47	3.37 ± 0.41	2.66 ± 0.10
Top-down control α (*Md* = 0.49)	0.52 ± 0.21	0.34 ± 0.12	0.71 ± 0.10
		**Right preference (*****n*** = **16)**	**Left preference (*****n*** = **15)**
Spatial laterality *w_*lat*_* (*Md* = 0.49)	0.49 ± 0.06	0.45 ± 0.04	0.54 ± 0.03

*Mean ± standard deviation are shown*.

*Md, Median value used to split the groups*.

**Table 3:** Pairwise correlations among TVA parameters.

**TVA parameters**	***C***	***K***	**α**
*C*			
*K*	0.18 (p = 0.328)		
α	0.18 (p = 0.343)	0.20 (*p* = 0.284)	
*w_lat_*	−0.09 (p = 0.636)	−0.03 (*p* = 0.873)	0.33 (*p* = 0.073)

*TVA parameters: C, visual processing speed; K, visual short-term memory storage capacity; α, top-down control; w_lat_, spatial laterality*.

### Selection of brain networks relevant for visual attention

**Figure 2:**
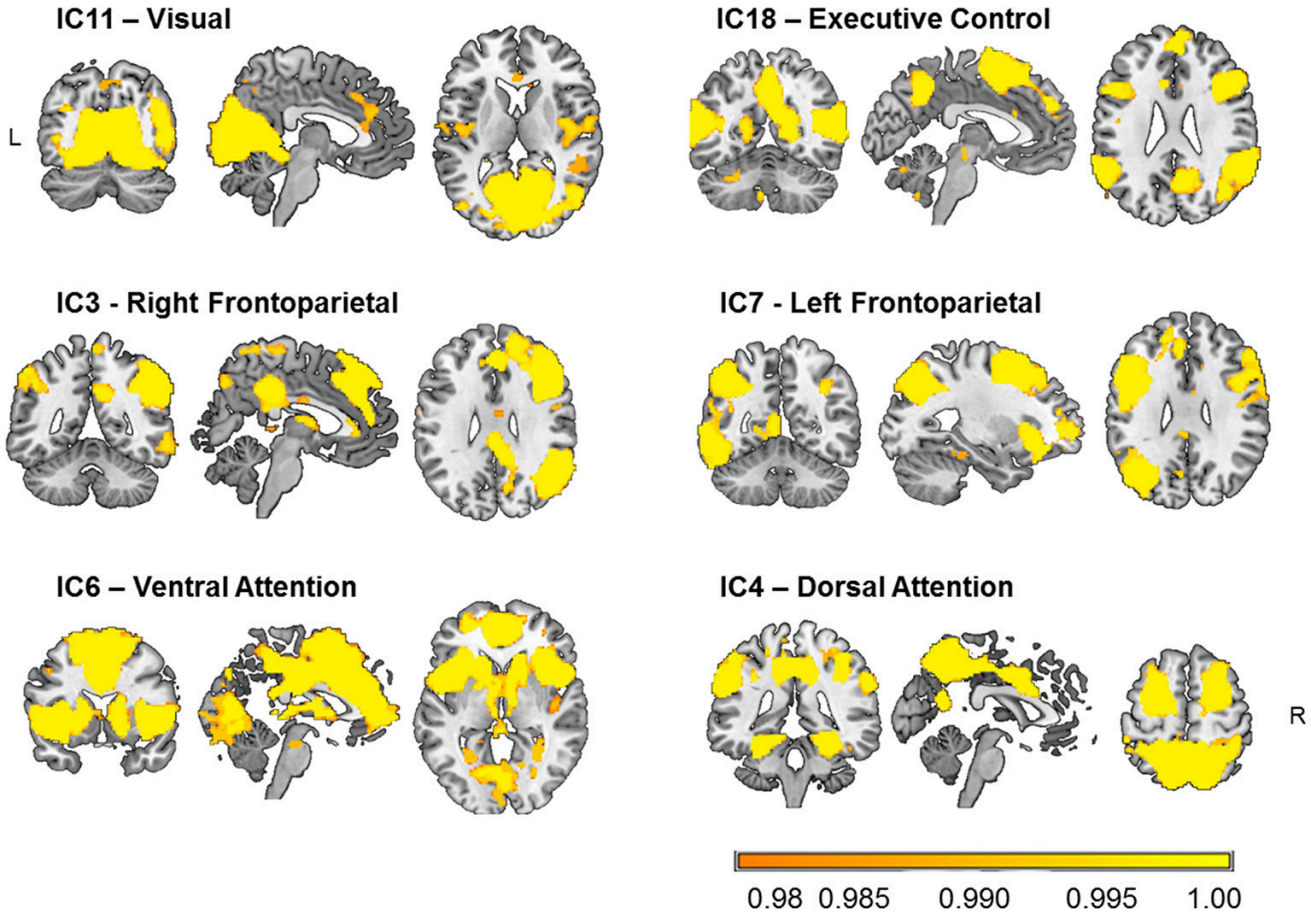
Visual attention-relevant brain networks selected from 20 components obtained from independent component (IC) analysis and dual regression of resting-state BOLD-fMRI data of 31 healthy young participants. The spatial maps represent voxels significantly belonging to each network (*p* < 0.05, FWE-corrected) and are overlaid onto an anatomical high-resolution brain-extracted template in MNI space (Holmes et al., 1998; Rorden and Brett, 2000; MRIcron). The labels just serve to identify them and follow conventional names given in the literature.

Six components that comprised occipital, lateral frontal and parietal, and limbic regions were selected as relevant for visual attention out of 12 functionally relevant components (Figure 2). These components were cross-correlated with the templates of Yeo et al. (2011) as well as with the ICA-based 28 network templates of Allen et al. (2011), and those with the highest coefficients were selected as networks (e.g., IC3: *r* = 0.57 with IC60 of Allen et al.; IC4: *r* = 0.40 with IC72; IC6: *r* = 0.49 with IC55; IC7: *r* = 0.34 with IC34; IC11: *r* = 0.43 with IC64; and IC18: *r* = 0.45 with IC27).

The components shown in Figure 2 comprise the IC11 or “visual” network, mainly encompassing occipital clusters on the lingual gyri and calcarine sulci, as well as clusters on the right middle frontal gyrus, and postcentral gyrus bilaterally. The IC18 or “executive control” network included temporal and frontal clusters bilaterally on the superior and middle temporal gyrus, and the inferior frontal and precentral gyri, as well as on the precuneus and calcarine sulci. The IC3 or “right frontoparietal” network comprised parietal clusters bilaterally on the inferior parietal lobule, superior and middle temporal gyrus, and inferior frontal gyrus, as well as on the left cerebellum and left calcarine sulcus. For IC7 or “left frontoparietal” network, clusters were observed mainly in left frontal and parietal areas, including the inferior frontal gyrus, intraparietal sulcus, as well as in the right cerebellum, and left and inferior temporal gyri. The IC6 or “ventral attention” network included bilateral frontoinsular regions such as the insula, anterior and middle cingulate cortex, middle frontal gyrus, as well as bilateral regions of the cerebellum, the thalamus, and the caudate nucleus, and of parieto-occipital areas. Finally, the IC4 or “dorsal attention” network was formed by bilateral parietal clusters of the precuneus, superior and inferior parietal lobules, supramarginal gyrus, as well as middle and inferior temporal, superior frontal, precentral, and fusiform gyri, and cerebellum.

### Intra-network differences in functional connectivity between performance groups

**Figure 3:**
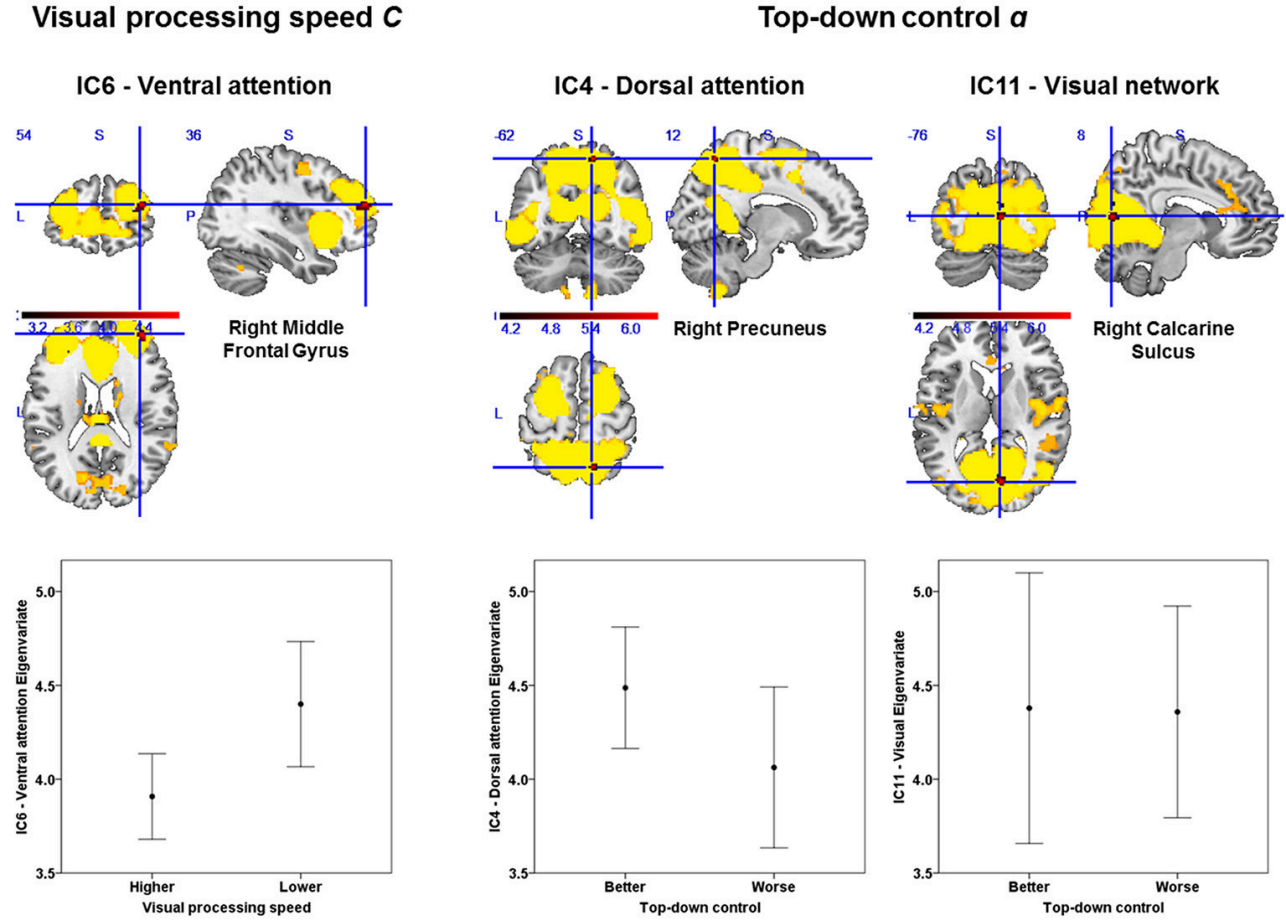
Group differences in intrinsic functional connectivity (FC). The group with higher visual processing speed *C* estimates showed lower FC of the right middle frontal gyrus within a ventral attention network (**left part**). The group with better top-down control α estimates showed both higher FC of the right precuneus within a dorsal attention (**middle part**) and lower connectivity of the right calcarine sulcus within a visual network (**right part**). Significant clusters (in red) are overlaid onto the respective group spatial maps of Figure 2 (in yellow). Below these maps, respective group differences can be observed with respect to the Eigenvariate or average FC of the networks. Error bars indicate standard error of the mean. Significant clusters have FWE-corrected *p* < 0.0083. Red bars show *t*-values (see also Table 4).

Based on our approach of median splits of a group of healthy participants, we observed voxel-wise intrinsic FC group differences in three particular attention-relevant brain networks (Table 4). With respect to capacity parameters, we found significant group differences for visual processing speed *C* in the ventral attention network, but no significant differences for VSTM capacity *K*. With regard to weighting parameters, we found significant group differences for top-down control α in the dorsal attention and visual networks. In addition, for spatial laterality *w*_*lat*_, we found significant differences in the right frontoparietal network—though this result did not survive Bonferroni correction at the network level (Table 4). In more detail, the group with relatively higher visual processing speed showed lower intrinsic FC of the right middle frontal gyrus in the ventral attention network (Figure 3). Moreover, the group with more efficient top-down control showed higher FC of the right precuneus in the dorsal attention network and lower FC of the right calcarine sulcus in the visual network.

**Table 4:** Group differences in intrinsic FC between subgroups defined according to TVA parameters.

**TVA parameter**	**Brain network**	**Peak brain area**	**Cluster size (voxels)**	**MNI coordinates (x, y, z) in mm**	***t* value**	***p* value**
*C*	Ventral attention	R middle frontal	60	36, 54, 20	4.79	0.008^*^
*K*	–	–	–	–	–	–
α	Dorsal attention	R precuneus	36	12, −62, 60	6.38	0.008^*^
	Visual	R calcarine sulcus	126	8, −76, 10	6.52	0.001^*^
*w_*lat*_*	Right frontoparietal	R Angular	55	34, −70, 50	5.08	0.038

*L, Left; R, Right*.

*All p values are corrected for Family-Wise Error (FWE)*.

* *Survive additional Bonferroni correction (p = 0.05/6 = 0.0083) at the network level*.

To account for possible differences in, for instance, noise levels between groups, we calculated the temporal signal-to-noise ratio of the realigned fMRI time series and repeated the analyses including the individual temporal signal-to-noise ratio as a covariate in the model. For the ventral attention and visual networks, the differences between groups remained the same [*t*_(23)_ = 4.74, *p* = 0.008, *k* = 60 voxels for the ventral attention network, and *t*_(23)_ = 6.49, *p* = 0.001, *k* = 129 voxels for the visual network; same cluster peaks for both as in Table 4]. For the dorsal attention network, the difference was slightly reduced, but still significant [*t*_(23)_ = 6.26, *p* = 0.013, *k* = 33 voxels]. Thus, group differences are unlikely attributable to systematic differences in signal quality. Differences in motion might still drive differences between groups. Given this, following (Smith et al., 2014), we checked whether performance groups differed in mean volume-to-volume head motion (e.g., Power et al., 2012), but we observed no significant differences: visual processing speed *C*: *t*_(29)_ = 0.82, *p* = 0.419; VSTM capacity *K*: *t*_(22.69)_ = 1.62, *p* = 0.113; top-down control: *t*_(29)_ = 0.47, *p* = 0.639; spatial laterality: *t*_(29)_ = 0.22, *p* = 0.826. As a further measure to ensure data quality (e.g., Smith et al., 2014), we checked participants with relatively extreme values in (a) the temporal signal-to-noise ratio, (b) the mean volume-to-volume head motion in mm, and (c) the proportion of outlier volumes. An extreme value was flagged if it was above or below the upper or, respectively, lower 5th percentile of the distribution of values in (a), (b), or (c). Two data points were flagged as extreme [< 9.39 in (a); > 0.26 in (b), and > 0.08 in (c)]. Excluding both participants slightly reduced the differences, which however remained significant (see Table 4 for comparison) [visual processing speed *C*: *t*_(22)_ = 5.45, *p* = 0.009, *k* = 59 voxels, peak = 38, 52, 18; top-down control α: dorsal attention network: *t*_(22)_ = 6.10, *p* = 0.022, *k* = 29, same peak; and visual network: *t*_(22)_ = 6.68, *p* < 0.0001, *k* = 165, peak: 8, −74, 10].

### Directionality of functional connectivity differences

Based on the results of previous research on structural connectivity and individual variability in attention functions (e.g., Chechlacz et al., 2015), we had expected high performers to show high, rather than low, intra-network FC. Consequently, we decided to also explore *inter*-network FC (i.e., among brain networks) to better understand the finding of a relatively lower intra-network FC (i.e., among brain regions within one network) in “high” compared to “low” performers. More specifically, we wanted to ascertain whether or not a higher *inter*-network FC is observed for the visual and ventral attention networks (i.e., those with lower *intra*-network FC) in high performers. Inter-network FC has been shown to vary among individuals, with this variation associated with attention performance (Kelly et al., 2008). Thus, we expected to find a difference also in inter-network FC between high and low performers. Moreover, the strength of the negative relationship between “task-positive” networks (such as the attention-relevant networks) and “task-negative” networks (such as the default-mode network, known to deactivate during task conditions) has been associated with more consistent behavioral performance (Kelly et al., 2008). Thus, we hypothesized a *positive* relationship among our “task-positive” networks for high performers. Finally, we determined whether a *high* inter-network FC is related to the *low* intra-network FC of the visual and ventral attention networks.

### Inter-network differences in functional connectivity between performance groups

Here, we examined whether the inter-network FC would be significantly higher for “high” than for “low” performers in the visual and ventral attention networks and whether a lower intra-network FC would correlate significantly with higher inter-network FC—as hypothesized.

**Figure 4:**
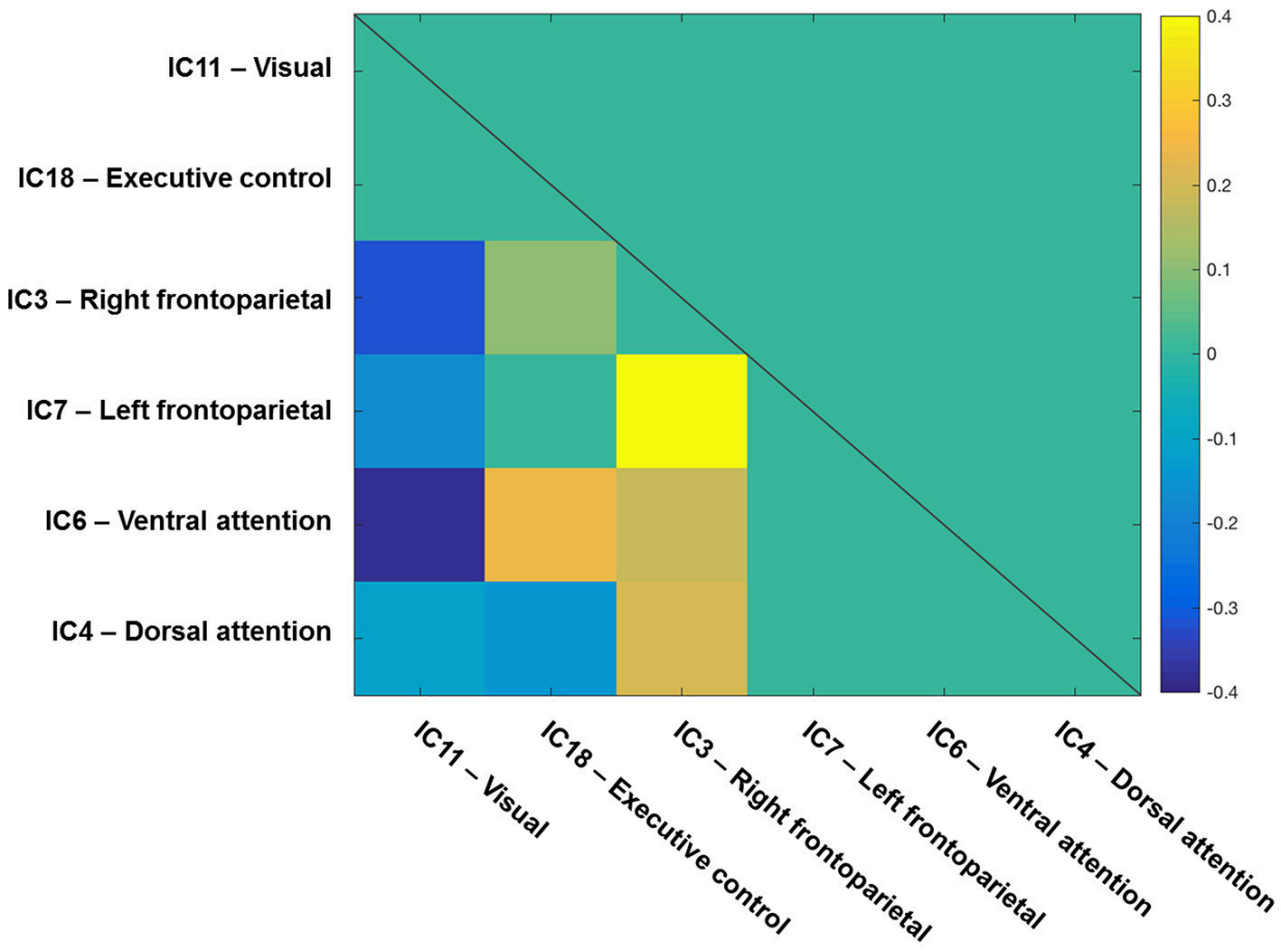
Inter-network functional connectivity (FC) among visual-attention relevant networks. One-sample *t*-test results (*q* < 0.05 FDR corrected for multiple comparisons) of the correlations among components on one side of a symmetrical matrix (below the diagonal line). Significant correlations are color-coded in warm (positive) and cool (negative) colors, whereas non-significant correlations are coded in turquoise. Spatial maps of components are depicted in Figure 2. The color bar shows mean Fisher r-to-z transformed values.

**Figure 5:**
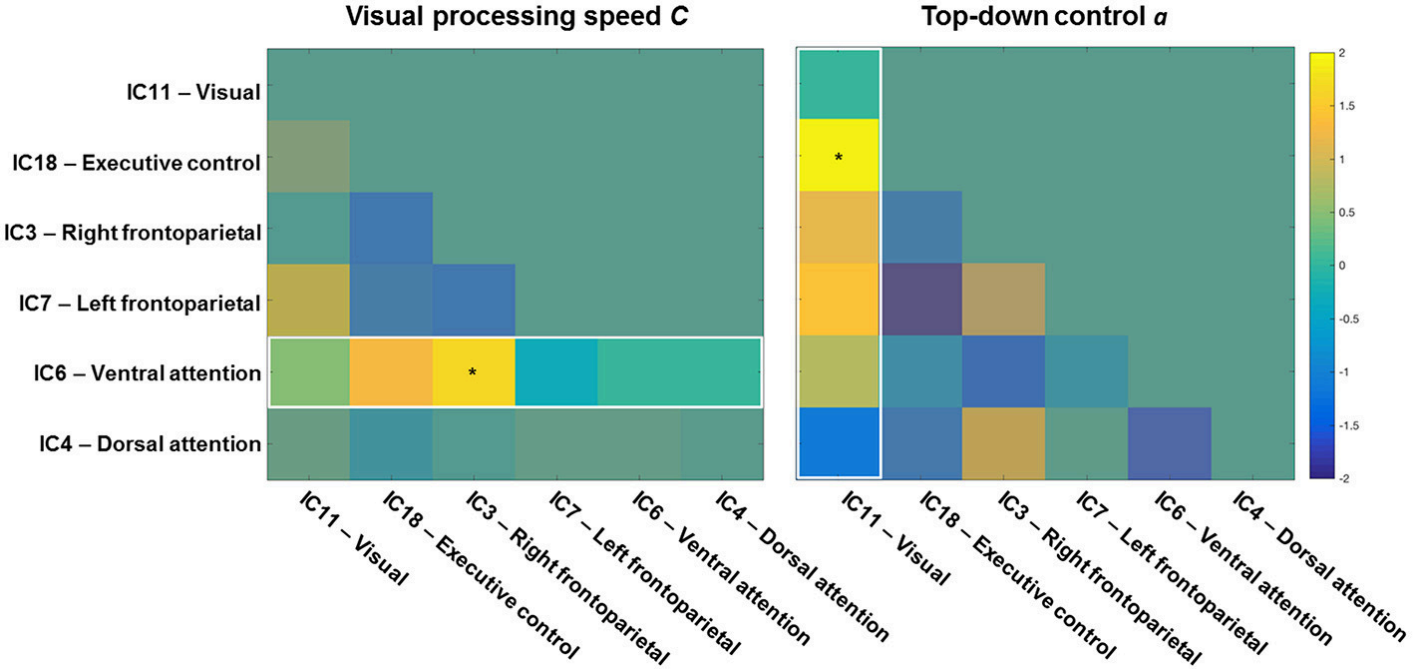
Visual processing speed (**left**) and top-down control (**right**) matrices showing *t*-values of high vs. low performance group differences. Higher inter-network functional connectivity (FC) values of the ventral attention (**left**), and visual (**right**) networks with the other networks were tested for the high performance group of speed and top-down control, respectively. The inter-network FC of the ventral attention network with the right frontoparietal network was significantly higher for the group with higher visual processing speed *C*. The inter-network FC of the visual network with the executive control network was significantly higher for the group with better top-down control α. The color bar shows *t* values (*df* = 29, high vs. low, *p* < 0.05).

To start with, the correlation matrix of the *Z* values (i.e., r-to-z transformation), averaged across the entire sample, is presented in Figure 4 to illustrate the inter-network FC. Next, Figure 5 depicts a group matrix for both visual processing speed *C* (left) and top-down control α (right), summarizing significant differences in inter-network FC between high and low performers. We only tested differences in the ventral attention network for visual processing speed, and in the visual network for top-down control (vector framed by white)—because, in both cases, the respective intra-network FC was lower for high compared to low performers. We found only the inter-network FC of the ventral attention network with the right frontoparietal network to be significantly higher in the group with higher visual processing speed *C* estimates (mean *Z*-values for high vs. low performers, 0.269 vs. 0.116, *t*_(29)_ = 1.685, *p* = 0.051, 1-tailed). For top-down control, only the inter-network FC of the visual network with the executive control network was significantly higher in the group with better (i.e., lower) top-down control α estimates (mean *Z*-values for better vs. poorer performers, 0.020 vs. −0.111, *t*_(29)_ = 1.895, *p* = 0.030, 1-tailed). These results, however, do not survive Bonferroni correction (i.e., *p* = 0.01).

The observed high inter-network FC in high performers could explain the low intra-network FC. To test for this possibility, we computed the correlation between intra-network FC in the ventral attention and visual networks and inter-network FC of these networks with the right frontoparietal and executive control networks, respectively (while controlling for the intra-network FC of the right frontoparietal and executive control networks, respectively). We found a trend toward a negative correlation for the ventral attention network (*r* = −0.28, *p* = 0.069), and a significant negative correlation (*r* = −0.31, *p* = 0.045) for the visual network. This pattern indicates that high inter-network FC could indeed explain the observed low intra-network FC in high performers.

## Discussion

We examined whether and how visual attention parameters derived from TVA-based model fitting that are assumed to represent latent traits underlying the individual efficiency of the visual selection process are mapped onto distinct brain networks obtained from intrinsic functional connectivity (FC). We divided the sample into groups of high and low performers for each relevant TVA parameter and compared the FC of networks that encompass cortical regions relevant for visual attention.

First, we found significant intra-network FC group differences for two TVA parameters. Participants with higher, vs. those with lower, visual processing speed exhibited lower FC of the right middle frontal gyrus within the ventral attention network. Furthermore, participants with more efficient, versus those with less efficient, top-down control exhibited higher FC of the right precuneus within the dorsal attention network and lower FC of the right calcarine sulcus within the visual network.

Second, we found that for those networks for which participants with superior attentional performance showed lower *intra*-network FC than those with inferior performance, the same participants also showed higher *inter*-network FC. More precisely, significantly higher inter-network FC was found for the ventral attention network with the right frontoparietal network in the group with higher compared to that with lower processing speed. For top-down control, significantly higher inter-network FC was found for the visual network with the executive control network in the group with more efficient compared to that with less efficient top-down control. Our results demonstrate for the first time a distinctive correspondence between particular visual attention parameters and FC of different brain networks. These results thus contribute to the evidence that, in healthy participants, relatively stable individual differences in attention functions are reflected in similarly stable differences in intrinsic FC.

### Visual attention capacity parameters

#### Visual processing speed *C* and the ventral attention network

Our finding of a linkage between visual processing speed and FC within the ventral attention network, and particularly in the right middle frontal gyrus, points to a role of this frontoparietal, limbic network for the rate of visual information uptake. As the ventral attention network has been previously documented to be relevant for tonic alertness (e.g., Sadaghiani et al., 2010; Sestieri et al., 2014; Coste and Kleinschmidt, 2016), the current result is in agreement with theoretical proposals (Bundesen et al., 2015) and empirical evidence (Matthias et al., 2009; Finke et al., 2010; Vangkilde et al., 2012; Petersen et al., 2017; Wiegand et al., 2017) for a close link between alertness and visual processing speed.

Further support for this link is provided by our additional finding (although uncorrected for multiple comparisons) of higher inter-network FC between the ventral attention and the right frontoparietal network. Right-sided brain regions have generally been implicated in the maintenance of an alert state under conditions without external warning cues (i.e., tonic alertness) and with increased time on task (i.e., vigilance) (e.g., Pardo et al., 1991; Sturm and Willmes, 2001). In healthy individuals, the right middle frontal gyrus has been shown to exhibit higher activity during maintenance of an alert state (Sturm et al., 1999), as well as higher spontaneous activity during high degrees of tonic alertness, as measured by pupil size changes (Schneider et al., 2016). Moreover, in patients with tonic alertness deficits following right-sided ventral lesions, tonic alertness training leads to an increase in the activity of the right middle frontal gyrus (Thimm et al., 2006). Similarly, stroke damage to areas in the right mid-frontal lobe, often involved in the neglect syndrome, can also produce deficits in sustained attention (Husain and Rorden, 2003). Finally, evidence from structural connectivity has also shown that the degree of right-side lateralization of the inferior fronto-occipital fasciculus is positively associated with visual processing speed in healthy young subjects (Chechlacz et al., 2015). In sum, in young healthy adults who process visual information faster, these frontoinsular and parietal networks that are important for tonic and phasic alertness, respectively, appear to be functionally well coupled.

According to TVA, visual processing speed represents the number of visual elements that can be categorized in a given unit of time (e.g., 1 s; Bundesen, 1990). This rate of encoding into VSTM depends on the strength of the sensory evidence, a perceptual decision bias, and the relative attentional weight of a specific object. In the neural interpretation of TVA, NTVA (Bundesen et al., 2005), the encoding speed is suggested to depend on both the number of cortical neurons representing the categorization and the firing rates of those neurons. More specifically, a perceptual decision bias determines how an object is categorized by changing the firing rate of the cortical neurons that code a particular feature (i.e., “pigeonholing”). The individual overall visual processing speed, parameter *C*, has been related, both theoretically and empirically, to alertness functions. For example, stimulant medication with methylphenidate and modafinil (Finke et al., 2010) as well as experimental manipulations enhancing phasic alertness (Matthias et al., 2009) have been shown to lead to an increase in this attentional capacity parameter. Recently, the effects of phasic alertness and temporal expectancy of upcoming stimuli were more formally integrated into the theory. More specifically, an enhancement of overall visual processing speed *C* was suggested, which would be given by a multiplicative upscaling of the activation, i.e., of the firing rates of all neurons coding the presented stimulus array by changes in perceptual bias (Vangkilde et al., 2012; Wiegand et al., 2017). Bias values have been proposed to derive from higher order areas (e.g., in frontal cortex) and, directly or indirectly, from the limbic system (Bundesen et al., 2005).

#### VSTM storage capacity *K*

One reason for our non-significant findings regarding this parameter might be the low variability of the *K* estimates and, thus, the lack of statistical power at the present sample size. Another reason might be the reliance of VSTM capacity on spatially organized sustained activity implemented via recurrent thalamocortical feedback loops (Bundesen et al., 2005), as supported by studies on the connectivity of thalamocortical fibers (Menegaux et al., 2017). Thus, future studies could examine inter-network thalamocortical FC in samples with greater variance in this parameter (e.g., in aging).

### Visual attention weighting parameters

#### Top-down control α and dorsal attention and visual networks

From a mechanistic perspective, the neural TVA suggests that top-down control is a selection bias, whereby higher “attentional weights” are assigned to objects that belong to a currently relevant category (e.g., red letters) (Bundesen et al., 2005). In the present study, we found that more efficient top-down control was linked with higher FC within the dorsal attention network, particularly in the precuneus. This result is in agreement with task-based neuroimaging studies (e.g., Wojciulik and Kanwisher, 1999; Hopfinger et al., 2000; Weissman et al., 2002; Giesbrecht et al., 2003; Vossel et al., 2016), which have also revealed a general role of dorsal parietal regions in the control of selective attention. Importantly, however, our results add to the existing evidence for a role of the precuneus in attentional top-down controlled, task-based selection that is *independent* of individual capabilities in spatial attentional selection or processing speed.

We found that more, versus less, efficient top-down control was associated with lower FC within the visual network, particularly in the calcarine sulcus. Importantly, lower FC within the visual network was significantly associated with higher inter-network FC of the visual network with the executive control network. Moreover, more efficient control was related to higher FC between the visual and the executive control networks, though this result did not survive Bonferroni correction. Thus, it appears that it is the degree of functional coupling of the visual network with the executive control network that might be relevant for the individual degree of efficiency of top-down control. This finding accords with the assumption of a critical role of the executive control network in the adaptive control of goal-directed selection (Dosenbach et al., 2007, 2008). Collectively, ours and previous findings suggest, in agreement with theoretical accounts of visual attention (Posner and Petersen, 1990; Desimone and Duncan, 1995; Bundesen et al., 2005), that the efficiency of top-down control is related to the degree of interaction between the executive control network generating attentional control signals and sensory structures that process visual information.

Of note, although we failed to find significant inter-network FC between the visual and the dorsal attention networks, our results do not imply a lack of functional interaction between the two. In fact, there are consistent reports of directed FC from the dorsal attention network regions to the visual network regions during tasks involving visuospatial attention (e.g., Corbetta and Shulman, 2002; Bressler et al., 2008; Spadone et al., 2015). Rather, they only suggest that higher intra-network FC in the dorsal attention network is by itself important for more efficient top-down control. In other words, the role of the intra-network FC for top-down control would be additional to that of the inter-network FC between the visual and executive control networks. This interpretation is in line with proposals according to which multiple cortical and non-cortical sources may be involved in top-down control if they carry information about the behavioral task goals (Gilbert and Li, 2013). On this view, our finding of significant inter-network FC of the visual network with the executive control network would not be surprising. In fact, the prefrontal cortex—a central component of the executive control network—has been shown to be a source of biasing signals in object-based attention (Baldauf and Desimone, 2014). Thus, rather than directly implying a lack of interaction between the visual and dorsal attention networks—or, put differently, an exclusivity of the executive control network for top-down control over the visual network—our results point to the relevance of all three networks.

#### Spatial laterality *w*_*lat*_

The lack of significant (Bonferroni corrected) group differences in any network for this parameter is not surprising in this sample of healthy young participants, given that no significant deviation from 0.5 in their *w*_*lat*_ values was present. In neurologically impaired samples, by contrast, parameter *w*_*lat*_ does exhibit high variance, such as in patients with mild cognitive impairment and mild Alzheimer's disease, in which significant spatial biases have been revealed (Redel et al., 2012; Sorg et al., 2012). Accordingly, studies on groups with more evident lateralized attentional performance might well reveal a relationship of parameter *w*_*lat*_ with FC.

### Visual attention functions in the “resting brain”

In mice, infra-slowly spontaneous neuronal fluctuations (i.e., 0.01–0.1 Hz) have been shown to underlie the intrinsic FC obtained from BOLD fMRI (Matsui et al., 2016). In humans, spontaneous slow cortical potentials (<0.5 Hz) measured with intracranial EEG have also been shown to be associated with intrinsic FC, where both have been proposed to reflect fluctuations of cortical excitability (He et al., 2008; Raichle, 2011). These fluctuations indicate spontaneous subthreshold depolarizations of the cortical neuronal membranes, which influences the level of activation of cortical neurons (Wu et al., 2008). If spontaneous fluctuations of cortical excitability do indeed influence attention continuously, their spatial patterns of coherence among brain regions and networks could be captured by intrinsic FC. In consequence, the differential spatial patterns obtained by FC could, then, distinguish among separable attention traits.

In support of such links, previous findings have suggested that particular functional interactions within (Markett et al., 2014; Rosenberg et al., 2016) and between (Kelly et al., 2008) spontaneously active functional networks relate to variability of performance in attention tasks. Here, we document that the differences among healthy individuals in attentional parameters that are assumed to reflect relatively stable capabilities or latent traits (e.g., Finke et al., 2005) correspond to the intra- and inter-network FC of particular functional networks.

### Functional implications and further issues

Healthy individuals differ in their ability to attend efficiently, and the TVA-based measurement provides for a systematic assessment of parameters expressing this variability (Habekost et al., 2014). While it is assumed that the different parameters reflect relatively stable capabilities of a given participant under stable environmental conditions (e.g., Finke et al., 2005), these capabilities might also change under particular circumstances. For example, the TVA parameters (Finke et al., 2010; Vangkilde et al., 2012) are influenced by certain pharmacological substances. Also, visual processing speed is enhanced by appropriate computerized training regimens (Schubert et al., 2015), whereas special populations such as patients with dyslexia (Stenneken et al., 2011), depression (Gögler et al., 2016), and schizophrenia (Gögler et al., 2017), exhibit reductions in visual processing speed. Furthermore, changes in attentional variability also occur during normal and pathological aging: healthy aging involves a slowing of visual processing (McAvinue et al., 2012; Habekost et al., 2013; Espeseth et al., 2014), and patients with mild cognitive impairment and the beginning of Alzheimer's disease reveal a staged decline of both visual processing speed (Bublak et al., 2011; Ruiz-Rizzo et al., 2017) and top-down control (Redel et al., 2012).

Of theoretical importance, documenting specific relationships between changes in attentional functioning and intrinsic FC can stimulate hypothesis-driven analyses as to the brain mechanisms that underlie the changes. For instance, in studies employing neurocognitive enhancement procedures, it could be tested whether FC in specific networks might serve as a treatment target or a predictor of treatment success. Further, as regards disorders characterized by attentional dysfunction, focusing on changes in the FC of specific networks might help to better understand the cause of different attentional syndromes and improve diagnosis and treatment. As for patient populations, the advantage of our approach derives from its ready applicability: as information on multiple visual attention traits and functional networks can be obtained with two simple psychophysical tasks and one short, easy fMRI session, it can be used in both cross-sectional and longitudinal studies. This renders the approach particularly attractive for studies of aging populations to examine more directly whether the increasing severity of attentional dysfunctions is related to FC changes within and between particular networks.

Our results highlight the relevance of particular functional networks for both visual attention capacity and weighting parameters. As a voxel-wise approach was used to identify the functional networks, differences were observed in specific regions within those networks. Note though that we do not consider those regions as being “responsible for” visual processing speed or top-down control, as the values associated with the voxels comprising those regions reflect their connectivity with a particular network (Beckmann et al., 2009; Smith et al., 2014), rather than their activity. Accordingly, we see them simply as clusters whose voxels reached statistical significance in this particular sample; at best, they allowed us to identify the relevant networks for visual attention functions. Furthermore, given that we relied on the group median to divide our sample of healthy young adults, we cannot make strong claims about “increased” or, respectively, “decreased” FC in our sample. We propose it would be more useful to examine whether the directionality of FC holds practical significance in terms of, for example, predicting the level of BOLD activity or connectivity during the actual performance of the whole- and partial-report tasks. Previous task-related fMRI studies have shown that individual differences in visual attention functions might not be reflected that much in differences in BOLD-evoked amplitudes (e.g., Gillebert et al., 2012), but rather in differential connectivity between regions (e.g., Vossel et al., 2016). Given the previous evidence, it might be interesting to examine, in future studies, the associations between “offline” (i.e., during rest) and “online” (i.e., during task) measures of FC with separate visual attention functions in order to establish the practical relevance of the directionality of FC.

### Limitations

In interpreting our results, several limitations must be taken into account. First, although oculomotor activity was not monitored during the tasks, systematic eye movements are unlikely because of the brief exposure durations in both the whole- and partial-report tasks. Second, previous work has shown that frame-to-frame motion can impact resting-state FC (e.g., Power et al., 2012). Although we relied on the power of ICA to remove the noise from the signal corresponding to functional networks (Beckmann and Smith, 2004; Zuo et al., 2010), a low-scale noise influence on FC measures remains possible. Thus, future studies ought to consider applying more stringent methods of head motion control such as scrubbing regressors even in samples of healthy young adults.

Finally, although we checked that our participants had not fallen asleep during the resting-state fMRI sequence, we cannot entirely exclude that some of them had, without being aware of it. However, possible micro-sleep is unlikely to have compromised our intrinsic FC measures for two reasons. First, previous research has shown that FC of both higher-order and primary sensory networks can be maintained during the transition from wakefulness to sleep (e.g., Larson-Prior et al., 2009). And second, while spatial changes within functional networks (i.e., decoupling of the default mode network) have been reported during deep sleep (e.g., Horovitz et al., 2009), it is improbable that our participants had reached deep sleep within the ~11 min duration of the resting-state fMRI sequence, as they were not sleep-deprived and reaching deep sleep in an unknown and noisy environment is difficult.

### Summary and conclusion

In sum, our study shows that visual attention functions correspond distinctively to the functional connectivity both within and between particular functional networks. Within networks, (i) higher visual processing speed was associated with lower functional connectivity within the ventral attention network; and (ii) more efficient top-down control was associated with higher functional connectivity within the dorsal attention network and lower functional connectivity within the visual network. Between networks, higher functional connectivity was observed between (i) the visual attention and right frontoparietal networks for higher visual processing speed; and (ii) the visual and executive control networks for more efficient top-down control. Finally, lower functional connectivity within a network might be explained by the higher functional connectivity between networks. To conclude, our results point to a distinctive network-based functional representation of separable visual attention functions, laying the basis for testing specific hypotheses about the neural mechanisms underlying these functions in aging or pathology.

## Author contributions

AR-R, KF, and CS designed the study; JN acquired the data; AR-R analyzed the data; AR-R, KF, and CS drafted the manuscript. All authors revised it critically and approved its final version.

### Conflict of interest statement

The authors declare that the research was conducted in the absence of any commercial or financial relationships that could be construed as a potential conflict of interest.
